# Accuracy of Artificial Intelligence-Based Technologies for the Diagnosis of Atrial Fibrillation: A Systematic Review and Meta-Analysis

**DOI:** 10.3390/jcm12206576

**Published:** 2023-10-17

**Authors:** Nikolaos Manetas-Stavrakakis, Ioanna Myrto Sotiropoulou, Themistoklis Paraskevas, Stefania Maneta Stavrakaki, Dimitrios Bampatsias, Andrew Xanthopoulos, Nikolaos Papageorgiou, Alexandros Briasoulis

**Affiliations:** 1 Department of Clinical Therapeutics, National and Kapodistrian University of Athens, 157 28 Athens, Greece; mirtosoti@med.uoa.gr (I.M.S.); abriasoulis@med.uoa.gr (A.B.); 2 Department of Internal Medicine, University Hospital of Patras, 265 04 Patras, Greece; themispara@hotmail.com; 3 Faculty of Medicine, Imperial College London, London SW7 2BX, UK; s.maneta-stavrakaki17@imperial.ac.uk; 4 Division of Cardiology, Columbia University, New York, NY 10027, USA; db3670@cumc.columbia.edu; 5 Department of Cardiology, University of Thessaly, 382 21 Larissa, Greece; andrewvxanth@gmail.com; 6 Barts Health NHS Trust, St Bartholomew’s Hospital, London EC1A 7BE, UK; n.papageorgiou@nhs.net

**Keywords:** atrial fibrillation, artificial intelligence, screening

## Abstract

Atrial fibrillation (AF) is the most common arrhythmia with a high burden of morbidity including impaired quality of life and increased risk of thromboembolism. Early detection and management of AF could prevent thromboembolic events. Artificial intelligence (AI)--based methods in healthcare are developing quickly and can be proved as valuable for the detection of atrial fibrillation. In this metanalysis, we aim to review the diagnostic accuracy of AI-based methods for the diagnosis of atrial fibrillation. A predetermined search strategy was applied on four databases, the PubMed on 31 August 2022, the Google Scholar and Cochrane Library on 3 September 2022, and the Embase on 15 October 2022. The identified studies were screened by two independent investigators. Studies assessing the diagnostic accuracy of AI-based devices for the detection of AF in adults against a gold standard were selected. Qualitative and quantitative synthesis to calculate the pooled sensitivity and specificity was performed, and the QUADAS-2 tool was used for the risk of bias and applicability assessment. We screened 14,770 studies, from which 31 were eligible and included. All were diagnostic accuracy studies with case–control or cohort design. The main technologies used were: (a) photoplethysmography (PPG) with pooled sensitivity 95.1% and specificity 96.2%, and (b) single-lead ECG with pooled sensitivity 92.3% and specificity 96.2%. In the PPG group, 0% to 43.2% of the tracings could not be classified using the AI algorithm as AF or not, and in the single-lead ECG group, this figure fluctuated between 0% and 38%. Our analysis showed that AI-based methods for the diagnosis of atrial fibrillation have high sensitivity and specificity for the detection of AF. Further studies should examine whether utilization of these methods could improve clinical outcomes.

## 1. Introduction

Atrial fibrillation (AF) is the most common arrhythmia in adults worldwide. AF can be completely asymptomatic, and often its initial presentation includes thromboembolic events, such as strokes. It is estimated that more than 25% of strokes are caused by previously asymptomatic atrial fibrillation. In most of the cases, the stroke could have been prevented if the atrial fibrillation had been detected earlier, and the patients were started on anticoagulation therapy [1].

Given that many of the complications are preventable, many screening strategies have been suggested [2,3]. Currently, the European Society of Cardiology (ESC) guidelines suggest opportunistic screening for people above 65 years old, and systematic screening for people > 75 years old or those with increased risk of stroke [3]. The recommended screening tools include pulse check, single-lead ECG > 30 s. or 12-lead ECG interpreted by a physician [3]. However, since AF is often paroxysmal, these screening methods result in many false negative results, and therefore their use is limited [2].

Over the last few years, mobile heath technology has been developing quickly [4]. So far, various mobile devices and smartwatches with AI algorithms have been developed to detect AF and they demonstrate high diagnostic accuracy against a gold standard (i.e., 12-lead ECG, single-lead ECG, telemetry, Holter monitor, or implantable cardiac monitor) [5,6,7].

So far, the two main technologies used by AI-based devices to automatically detect AF are the photoplethysmography (PPG) and the single-lead ECG. The former is a photoelectric method that measures changes in blood volume in the peripheral vessels. PPG devices consist of a light source and receptor, and based on the reflected light can detect changes in the blood volume. These changes can be captured in a PPG trace which is then interpreted by an AI algorithm [8,9]. The single-lead ECG methods consist of a portable or wearable device which can record a single-lead ECG trace. To complete this assessment, the individual is asked to keep two parts of their body (e.g., wrist and finger or two fingers, etc.) in touch with the device for a pre-determined time. The recording is then transmitted to an AI application for interpretation [10,11,12]. These AI methods classify their recordings as “possible AF”, “normal” or “no AF”, “undiagnosable/unclassified”, or “error” [11,12].

Compared to the conventional methods, AI-based devices for the diagnosis of AF are widely available, easy to use, and offer prolonged monitoring times, which increase the chances of detecting paroxysmal episodes of AF [12]. If accurate, they can also accelerate the decision-making process by the physicians, who could use these data without the need to wait for further time-consuming investigations. In addition, single-lead ECG devices can save the ECG tracings, which can then be reviewed by a physician.

On the other hand, the rapid increase in uncertified devices and applications can lead to many false results. This can cause stress to the patients, unnecessary treatments and investigations, and a cost burden for the health care systems [12]. Also, single-lead ECGs are conducted by untrained individuals rather than trained health care professionals, which can result in poor quality tracings and thus unreliable outcomes [12].

The aim of our study is to provide a systematic review and meta-analysis of the diagnostic accuracy of all the available AI-based methods for the diagnosis of atrial fibrillation.

## 2. Materials and Methods

This systematic review–metanalysis was designed and conducted based on the Preferred Reporting Items for Systematic Reviews and Meta-Analyses (PRISMA) guidelines [13]. PROSPERO registration: https://www.crd.york.ac.uk/prospero/display_record.php?RecordID=357232 accessed on 10 July 2023 [14].

### 2.1. Inclusion and Exclusion Criteria

We included: (1) diagnostic studies with a cohort or case–control design, (2) studies conducted in adults 18 years old and above, (3) studies which tested AI-based devices to detect AF, (4) studies which used an acceptable reference standard interpreted via a healthcare professional, including 12-lead ECG, 6-lead ECG, single-lead ECG, 3-lead Holter monitor and telemetry, (5) studies that provided true positive, true negative, false positive, and false negative results or provided enough data to calculate them, (6) studies in which unclassified/unreadable results by the devices were reported separately.

Exclusion criteria included: (1) conference abstracts or studies without available full text, (2) studies published in a language other than English, (3) studies that only provided measurement-based instead of individual-based results, (4) studies that validated novel devices without automated interpretation, (5) studies in which the reference standard test was not completed in all the participants.

Unclassified results are the ones that could not be classified by the automated algorithm as AF or not AF. Unreadable results are the ones that could not be interpreted by the automated algorithm, e.g., poor quality or short tracings.

### 2.2. Data Sources and Search Strategy

To identify all the relevant studies, we searched the databases: (1) PubMed, (2) Embase, (3) Cochrane Library, and (4) Google Scholar. In addition, we conducted a manual search for further eligible studies.

The search in PubMed was undertaken on 31 August 2022, in Cochrane Library and Google Scholar on 3 September 2022 and in the Embase database on 15 October 2022.

The search strategy we used was:

((ai OR artificial intelligence OR machine learning OR ml OR deep learning OR neural network OR wearables OR smartwatches OR wearable OR smartwatch OR applewatch OR alivecor OR iECG) AND (diagnosis OR diagnosing OR detection OR detect OR detecting) AND (af OR atrial fibrillation OR afib OR arrhythmia OR svt OR supraventricular tachycardia OR atrial flutter OR tachycardia)).

The search strategy was created by the first author (NMS), reviewed by a second member of the team (IMS), and approved by the supervising professor (AB).

### 2.3. Screening

The identified citations were imported in the web application Covidence, which is endorsed by the Cochrane Collaboration for the conduction of systematic reviews [15]. The screening was performed by two independent and blinded researchers (NMS and IMS). Initially, duplicates were removed either automatically by the Covidence web app, or, less frequently, manually by the researchers. Following that, we screened the studies by reading the title and abstract, and then, for the selected studies, we performed a full-text review. Studies that met our inclusion and exclusion criteria were selected. In case of disagreement, the 2 researchers discussed until an agreement was reached.

### 2.4. Data Extraction

Data extraction was executed in Microsoft Excel, version 16.69. In case of uncertainty, a second researcher was asked to extract the data for the study in question, which was then discussed. In addition, when data calculation was impossible, the authors were contacted. If this was impossible, the study was reviewed by the second researcher before exclusion. For all the included studies, we extracted data including among others: the first author, the year of publication, the setting (inpatient vs. outpatient), the study design, the name of the device, the type of AI algorithm, the duration of the index test, the reference standard, basic demographics, true positive and negative results, false positive and negative results, and unclassified and unreadable results.

### 2.5. Assessment of Risk of Bias and Applicability

For the assessment of risk of bias and applicability, we used the quality assessment of diagnostic accuracy studies—2 (QUADAS-2) tool, which is recommended by the Cochrane Collaboration and the U.K. National Institute for Health and Care Excellence [16]. We assessed each study in 4 domains (1) selection of participants, (2) index test, (3) reference standard, (4) flow and timing. For each study, we also assessed the first 3 domains regarding its applicability. We used predetermined signaling questions tailored to our review. The assessment of risk of bias and applicability was performed by the main researcher (NMS).

### 2.6. Statistical Analysis

Data synthesis was conducted separately for the two main types of technology, photoplethysmography (PPG) and single-lead ECG. For the studies that tested technologies other than the above two, we did not perform a quantitative analysis due to lack of sufficient data, however, we describe their results. As effect measures of diagnostic accuracy, we used sensitivity and specificity. For the unclassified/unreadable results, we did not perform a quantitative analysis, however we describe them separately for each group. To present the unclassified/unreadable outcomes, we used their percentages out of total results as the effect measure. Studies that tested more than one device/technology are included as separate studies. We performed subgroup analysis on the PPG (inpatients vs. outpatients) and single-lead ECG groups (inpatients vs. outpatients and duration of index test).

To calculate our summary values and create the graphical interpretations, we used the mada package in R, version 4.2.3 (which uses the bivariate model of Reitsma, which is equivalent with the HSROC of Rutter and Gatsonis when covariates are not used). Also, we used the interactive online application MetaDTA, version 2.0 [17]. For the data synthesis, we used the random effects methodology due to the expected clinical heterogeneity among the studies. Due to the lack of a gold standard for the assessment of heterogeneity in diagnostic accuracy studies, we used the Zhou and Dendukuri approach, which considers the correlation between sensitivity and specificity for the calculation of I^2^ [18].

## 3. Results

### 3.1. Study Selection

**Figure 1:**
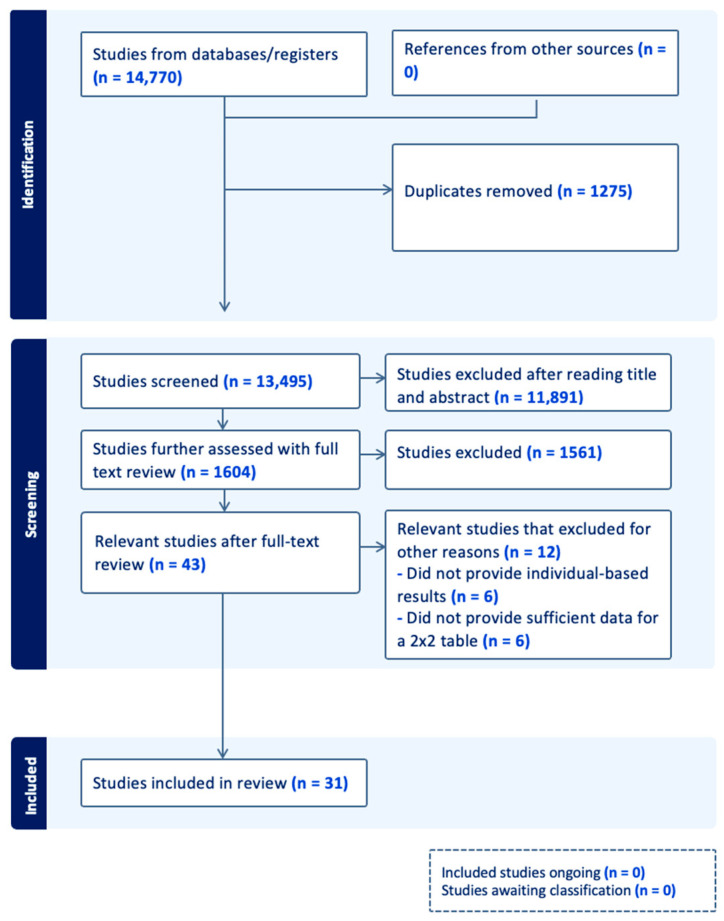
Flow chart (n: number).

The flowchart (Figure 1) illustrates our study selection process. We identified 14,770 studies from which 43 were selected. From those, 12 studies were excluded in a later stage. Six of them were excluded because they only provided measurement-based, and not patient-based, results [19,20,21,22,23]. The remaining six studies were excluded because they either did not provide enough data or we were unable to communicate with the authors to provide data for analysis [24,25,26,27,28,29]. In the end, 31 studies were included in our analysis (Figure 1).

### 3.2. Diagnostic Performance of Photoplethysmography (PPG) Devices

#### 3.2.1. Study Characteristics of PPG Studies

We identified 12 diagnostic accuracy studies that tested PPG devices for the diagnosis of atrial fibrillation. Their characteristics are summarized in Table 1. Nine studies had a case–control design and three had a cohort design. The total number of participants was 4579. The smallest study included 51 patients [30], and the biggest, 1057 [31]. Five studies were conducted in an outpatient setting [11,31,32,33,34], five studies in an inpatient setting [30,35,36,37,38] and two studies in both settings [39,40]. Eight studies [30,31,33,36,37,38,39,40] used smartwatches, three studies [11,32,35] used mobile phones and one study [34] tested the technology of a remote PPG with the use of industrial camera. Seven studies tested the devices for up to 5 min [11,32,35,36,37,39,40], and the rest tested the devices from 10 min to up to 1 week [30,31,33,34,38]. Three studies [36,37,38] tested more than one device/technology, therefore each one was included as a separate study.

#### 3.2.2. Assessment of Risk of Bias and Applicability of PPG Studies

**Figure 2:**
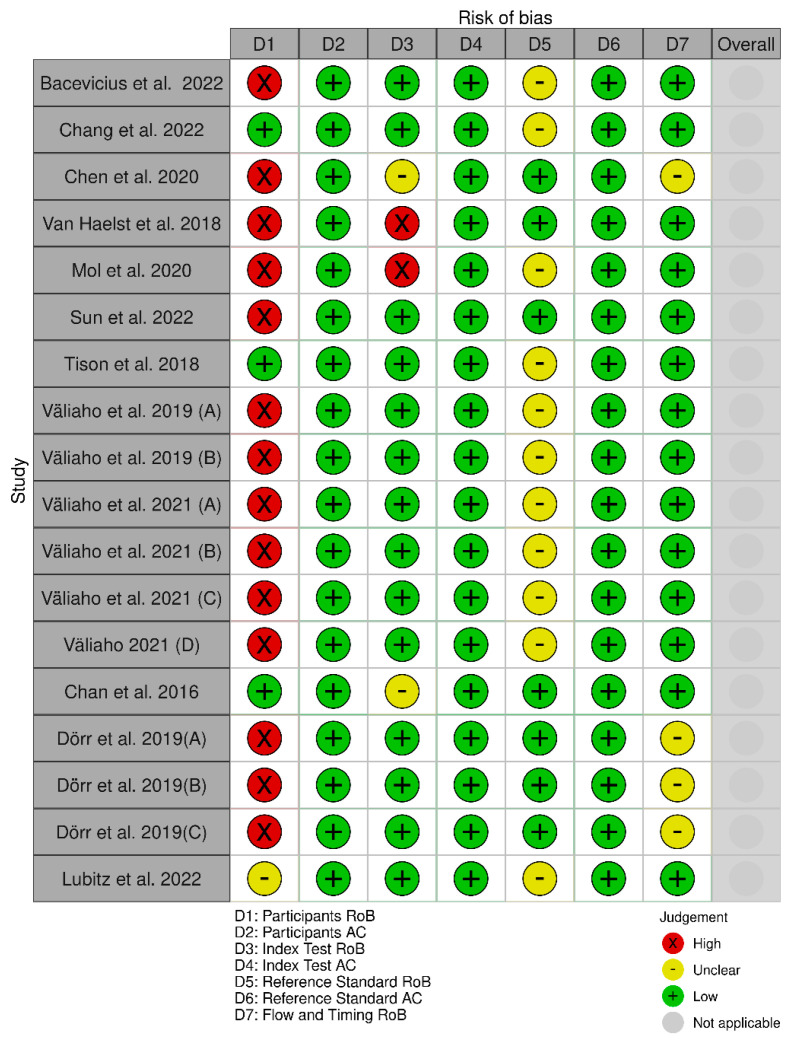
Assessment of risk of bias and applicability of the PPG studies. (Väliaho et al., 2019 (A): testing the AFEvidence algorithm; Väliaho et al., 2019 (B): testing the COSEn algorithm; Väliaho et al., 2021 (A): testing device performance when time interval between every measurement is 10 min; Väliaho et al., 2021 (B): testing device performance when time interval between every measurement is 20 min; Väliaho et al., 2021 (C): testing device performance when time interval between every measurement is 30 min; Väliaho et al., 2021 (D): testing device performance when time interval between every measurement is 60 min; Dörr et al., 2019 (A): testing performance of device when recording for 1 min; Dörr et al., 2019 (B): testing performance of device when recording for 3 min; Dörr et al., 2019 (C): testing performance of device when recording for 5 min).

Fourteen studies [11,34,35,36,37,38,39,40] were deemed high risk of bias in the participants’ domain, and two studies [11,35] in the index test domain. The rest were deemed either low or unclear risk of bias (Figure 2). The studies were low in risk regarding their applicability (Figure 2).

#### 3.2.3. Data Synthesis of the PPG Studies

**Figure 3:**
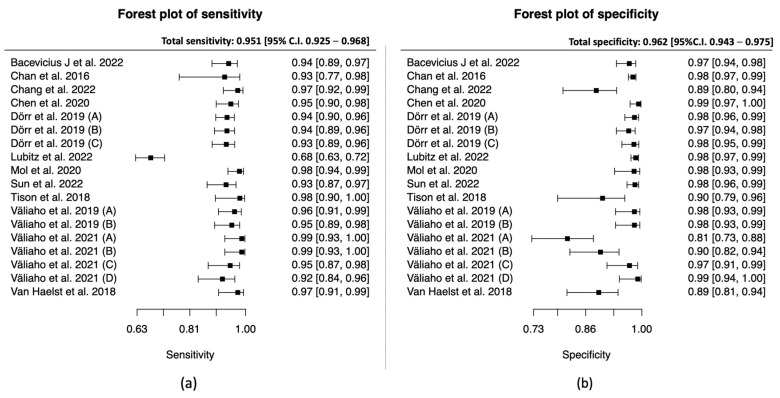
PPG group: (**a**) Forest plot of sensitivity; (**b**) forest plot of specificity. (Väliaho et al., 2019; (A): testing the AFEvidence algorithm, Väliaho et al., 2019; (B): testing the COSEn algorithm, Väliaho et al., 2021; (A): testing device performance when time interval between every measurement is 10 min, Väliaho et al., 2021; (B): testing device performance when time interval between every measurement is 20 min, Väliaho et al., 2021; (C): testing device performance when time interval between every measurement is 30 min, Väliaho et al., 2021; (D): testing device performance when time interval between every measurement is 60 min, Dörr et al., 2019; (A): testing performance of device when recording for 1 min, Dörr et al., 2019; (B): testing performance of device when recording for 3 min, Dörr et al., 2019; (C): testing performance of device when recording for 5 min).

**Figure 4:**
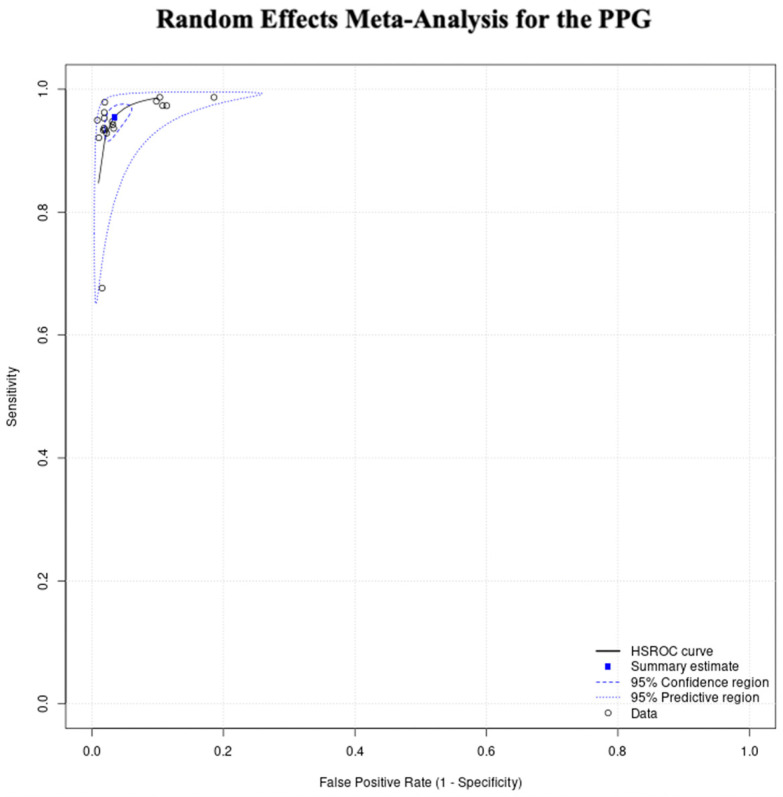
PPG group random effects meta-analysis.

The total sensitivity for the diagnosis of atrial fibrillation in the PPG group was 95.1% (95% C.I. 92.5–96.8%), the specificity was 96.2% (95%C.I. 94.3–97.5%), the area under the curve (AUC) for the SROC curve was 0.983 and the partial AUC was 0.961. The I^2^ was 12.5% (Figure 3 and Figure 4).

Among the studies, the AF prevalence was found to be between 2.5% and 57%, with a median prevalence of 44%. Based on these data, we used the total sensitivity and specificity to calculate the predictive false results in 1000 patients, by using different prevalence values. For prevalence of 5%, PPG devices would have resulted in 47 (95% C.I. 30–71) false positive results and 2 (95% C.I. 1–3) false negative results in 1000 patients. For the median prevalence of our studies, 44%, PPG devices would have resulted in 27 (95% C.I. 18–42) false positive results and 17 (95% C.I. 11–25) false negative results in 1000 patients. For a high prevalence of 60%, PPG devices would have resulted in 20 (95% C.I. 13–30) false positive results and 23 (95% C.Ι. 15–34) false negative results in 1000 patients.

#### 3.2.4. Subgroup Analysis (Inpatients vs. Outpatients) of the PPG Studies

**Figure 5:**
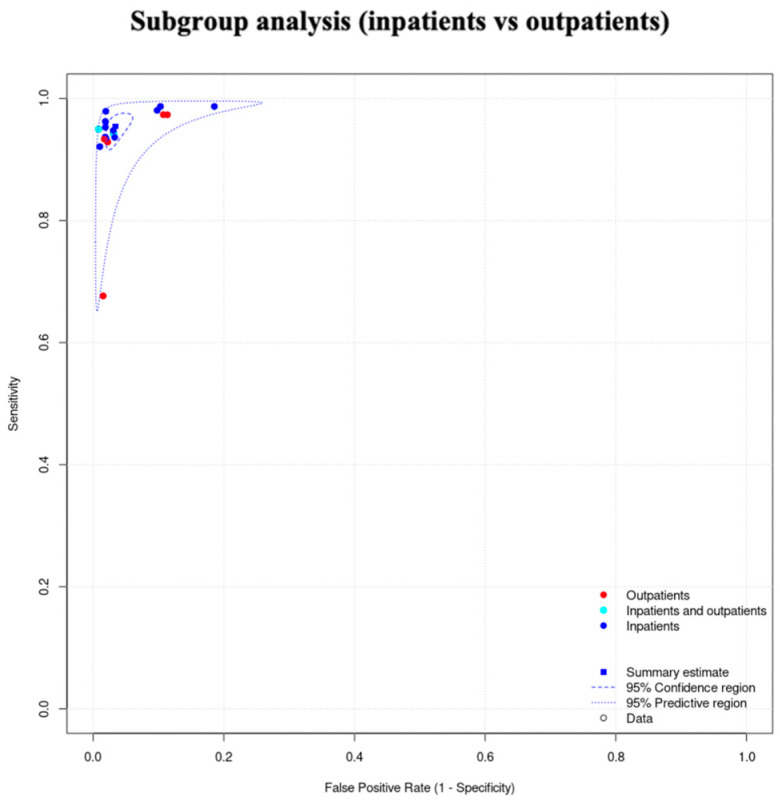
Subgroup analysis of the PPG studies (inpatients vs. outpatients).

We did not proceed to a formal subgroup analysis for the PPG studies due to the low number of studies per subgroup, but also because we did not observe clusters in this subgroup’s SROC curve (Figure 5).

#### 3.2.5. Unclassified Unreadable Results of the PPG Studies

We found significant heterogeneity among the studies, regarding the unclassified/unreadable results. The reported unclassified/unreadable results ranged from 0% at the lowest [30,31,32,33,38] to 43.2% at the highest (Table 1) [37].

### 3.3. Diagnostic Performance of Single-Lead ECG Devices

#### 3.3.1. Study Characteristics of the Single-Lead ECG Studies

During our search, we identified 22 diagnostic accuracy studies that tested single-lead ECG devices with AI-based algorithms for the diagnosis of atrial fibrillation. Their characteristics are summarized in Table 1. Eleven studies had cohort design [11,32,40,42,45,46,48,50,54,56,57] and the rest had case–control design [10,37,41,43,44,47,49,51,52,53,55]. The total number of participants was 6597. The smallest study included 50 patients [57] and the biggest study included 1013 patients [32]. Eleven studies were conducted in inpatient setting [37,41,43,45,46,50,51,52,54,55,57], 6 studies were conducted in outpatient setting [10,11,32,48,49,56], 2 studies were conducted in both settings [40,44] and in 3 studies the setting was not clear [42,47,53]. Eight studies tested smartwatches [10,40,42,43,44,47,50,53] and the rest of studies tested other devices [11,32,37,41,45,46,48,49,51,52,54,55,56,57]. Five studies [10,46,47,51,53] tested more than one device/technology, and therefore each one included as a separate study.

#### 3.3.2. Assessment of Risk of Bias and Applicability of the Single-Lead ECG Studies

**Figure 6:**
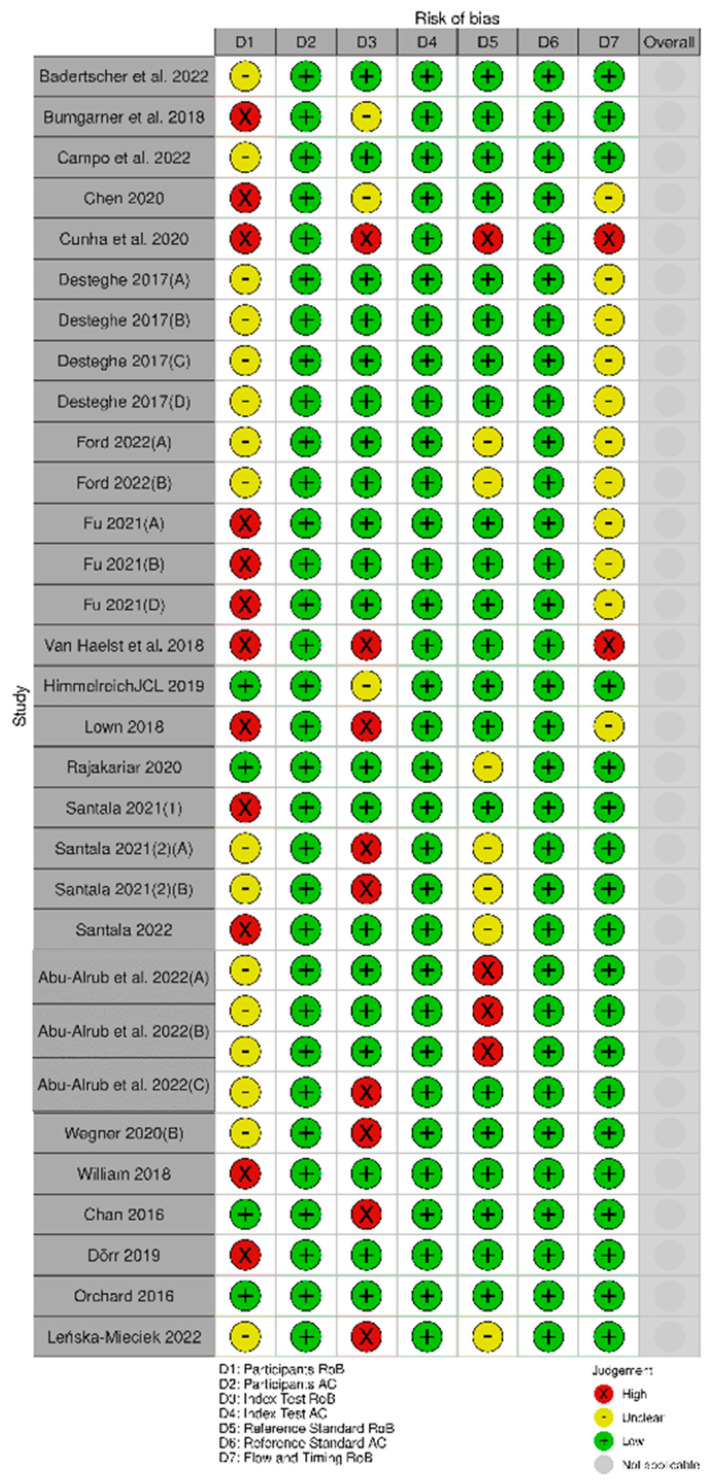
Assessment of risk of bias and applicability of the single-lead ECG studies. (Desteghe et al., 2017(A): testing the AliveCor in the cardiology ward population; Desteghe et al., 2017 (B): testing the MyDiagnostick in the cardiology ward population; Desteghe et al., 2017 (C): testing the AliveCor in the geriatric ward population; Desteghe et al., 2017 (D): testing the MyDiagnostick in the geriatric ward population; Ford et al., 2022 (A): testing the Apple Watch 4; Ford et al., 2022 (B): testing the KardiaBand; Fu et al., 2021 (A): testing the device in supine position, Fu et al., 2021 (B): testing the device in upright position, Fu et al., 2021 (C): testing the device after individuals climbed to the 3rd floor; Santala et al., 2021 (1): published in October 2021, Santala et al., 2021 (2) (A): published in May 2021 and testing the device between the palms, Santala et al., 2021, (2) (B): published in May 2021 and testing the device in the chest; Abu-Alrub et al., 2022(A): testing the Apple Watch 5, Abu-Alrub et al., 2022 (B): testing the Samsung Galaxy Watch Active 3, Abu-Alrub et al., 2022 (C): testing the Withings Move ECG; Wegner et al., 2020 (A): testing the lead I; Wegner et al., 2020 (B): testing the novel parasternal lead).

Twelve studies [11,37,40,41,43,45,47,49,52,55] were deemed to be at high risk of bias in the participants’ domain, nine studies [11,32,45,49,51,54,57] in the index test domain, four studies [45,53] in the reference standard domain, and two studies [11,45] were deemed to be at high risk of bias in the flow and timing domain (Figure 3). The studies were low in risk regarding their applicability (Figure 6).

#### 3.3.3. Data Synthesis of the Single-Lead ECG Studies

**Figure 7:**
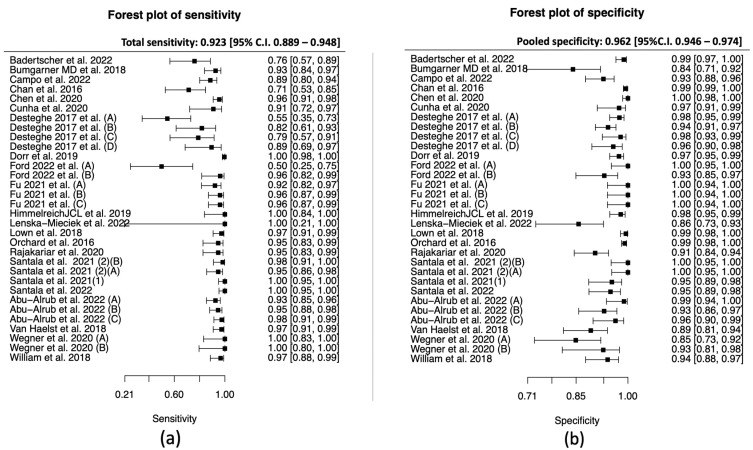
Single-lead ECG group: (**a**) forest plot of sensitivity, (**b**) forest plot of specificity. (Desteghe et al., 2017 (A): testing the AliveCor in the cardiology ward population; Desteghe et al., 2017 (B): testing the MyDiagnostick in the cardiology ward population; Desteghe et al., 2017 (C): testing the AliveCor in the geriatric ward population; Desteghe et al., 2017 (D): testing the MyDiagnostick in the geriatric ward population; Ford et al., 2022 (A): testing the Apple Watch 4, Ford et al., 2022 (B): testing the KardiaBand; Fu et al., 2021 (A): testing the device in supine position, Fu et al., 2021 (B): testing the device in upright position, Fu et al., 2021 (C): testing the device after individuals climbed to the 3rd floor; Santala et al., 2021 (1): published in October 2021, Santala et al., 2021 (2) (A): published in May 2021 and testing the device between the palms, Santala et al., 2021 (2) (B): published in May 2021 and testing the device in the chest; Abu-Alrub et al., 2022 (A): testing the Apple Watch 5, Abu-Alrub et al., 2022 (B): testing the Samsung Galaxy Watch Active 3, Abu-Alrub et al., 2022 (C): testing the Withings Move ECG, Wegner et al., 2020 (A): testing the lead I, Wegner et al., 2020 (B): testing the novel parasternal lead).

**Figure 8:**
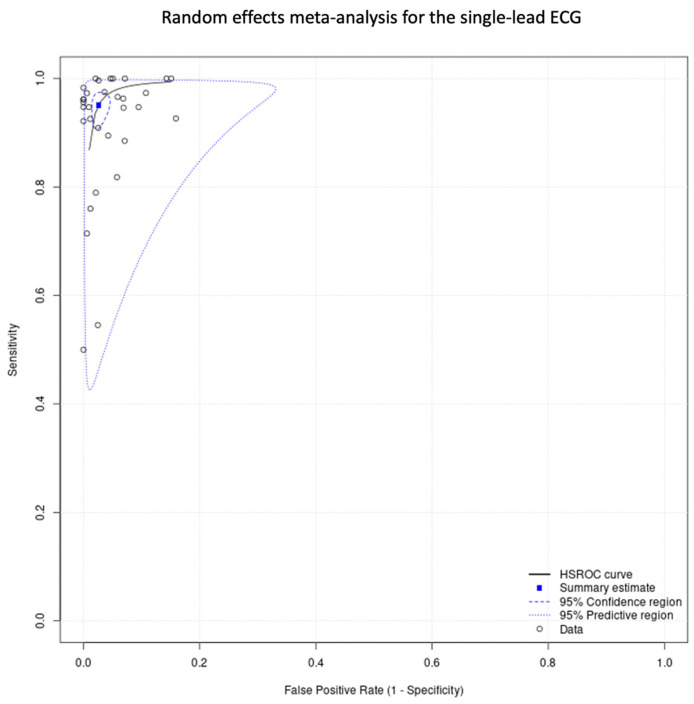
Single-lead ECG group: random effects meta-analysis.

The total sensitivity for the detection of atrial fibrillation by using single-lead ECG was 92.3% (95% C.I. 88.9–94.8%), the specificity was 96.2% (95%C.I. 94.6–97.4%), the area under the curve (AUC) for the SROC curve was 0.979, and the partial AUC was 0.939. The I^2^ was 9.2% (Figure 7 and Figure 8).

Among the studies, the AF prevalence was found to be between 2% and 61%, with median prevalence 31%. Based on these data, we used the total sensitivity and specificity to calculate the predictive false results in 1000 patients, by using different prevalence values. For a prevalence of 5%, a single-lead ECG device would have resulted in 73 (95% C.I. 49–106) false positive results and 2 (95% C.I. 1–3) false negative results in 1000 patients. For the median prevalence of our studies, 31%, a single-lead ECG device would have resulted in 53 (95% C.I. 36–77) false positive results and 12 (95% C.I. 8–17) false negative results in 1000 patients. For a high prevalence of 60%, a single-lead ECG device would have resulted in 31 (95% C.I. 21–44) false positive results and 23 (95% C.Ι. 16–32) false negative results in 1000 patients.

#### 3.3.4. Subgroup Analysis (Inpatients vs. Outpatients) of the Single-Lead ECG Studies

We conducted a subgroup analysis according to the setting. In this analysis, we did not include either the studies in which the setting was not clear, or the ones that included both inpatients and outpatients.

**Figure 9:**
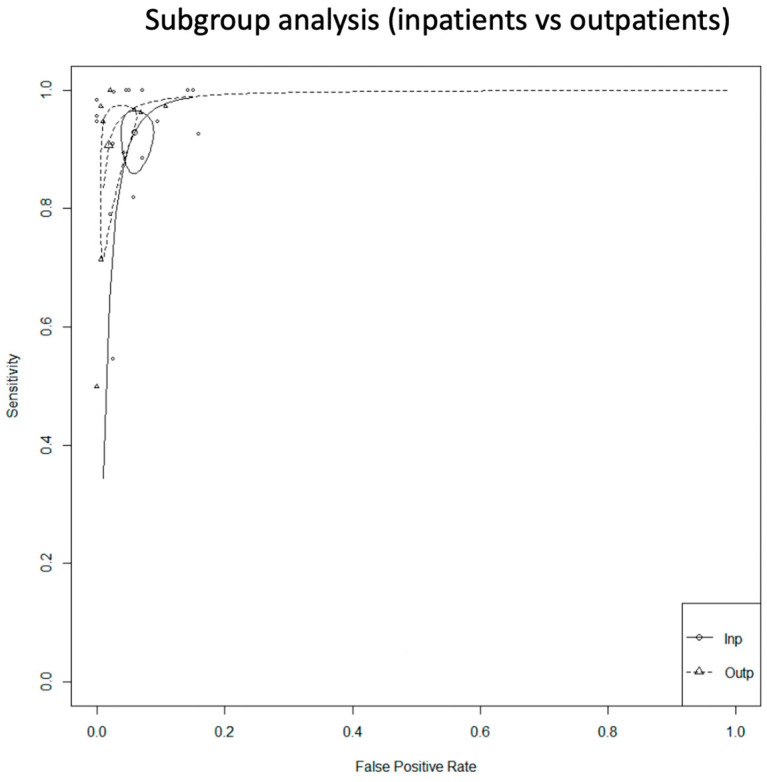
Single-lead ECG group: subgroup analysis (inpatients vs. outpatients).

For the inpatients, the total sensitivity was 92.9% (95% C.I. 87.6–96) and the specificity was 94.2% (95% C.I. 91.8–95.9). The AUC was 0.974 and the partial AUC was 0.898. The I^2^ was 14.4%. For the outpatients, the total sensitivity was 90.7% (95% C.I. 76.8–96.6) and the specificity was 98.1% (95% C.I. 95.1–99.3). The AUC was 0.983 and the partial AUC was 0.949. The I^2^ was 26.9%. Although the sensitivity was higher in the inpatient group, the specificity was higher in the outpatients. However, the 95% confidence intervals were overlapping. In addition, there was a difference in I^2^ between the subgroups. In the inpatient group, the I^2^ was 14.4%, and in the outpatient group it was 26.9% (Figure 9).

#### 3.3.5. Subgroup Analysis (Duration of Index Test) of the Single-Lead ECG Studies

**Figure 10:**
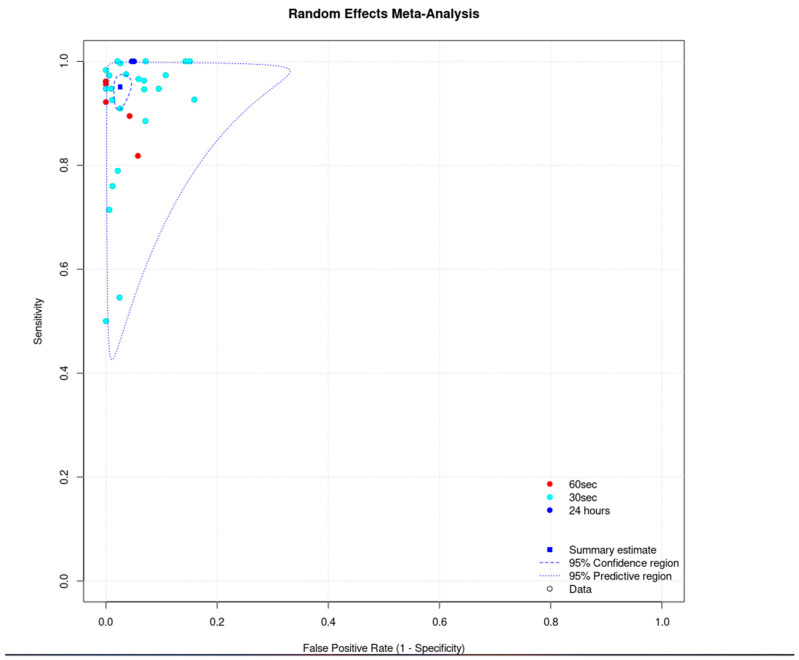
Single-lead ECG group: subgroup analysis (duration of index test).

We did not proceed to a formal subgroup analysis regarding the duration of the index test since most of the studies used it for 30 s (Figure 10).

#### 3.3.6. Unclassified/Unreadable Results of the Single-Lead ECG Studies

Regarding the unclassified/unreadable results, we also identified significant heterogeneity in the single-lead ECG group. The reported unclassified/unreadable results ranged from 0% the minimum [32,41,46,52,57] to 38% the maximum (Table 1) [54].

### 3.4. Diagnostic Performance of Technologies Other Than PPG or Single-Lead ECG

**Figure 11:**
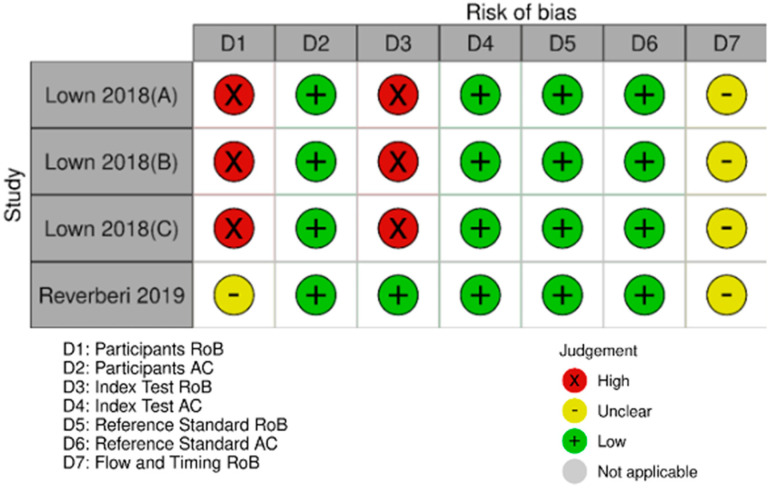
Miscellaneous technologies—assessment of risk of bias and applicability. (Lown et al., 2018 (A): testing the Watch BP, Lown et al., 2018 (B): testing the Polar H7 device, Lown et al., 2018 (C): testing the Bodyguard 2 device; D: domain; RoB: risk of bias; AC: applicability).

As mentioned earlier, some of the studies tested technologies other than PPG and single-lead ECG. Due to there only being a few studies, we did not proceed to quantitative synthesis, but we have described them separately. Their characteristics are summarized in Table 1 and Figure 11.

The study of Lown et al., 2018 [49], apart from single-lead ECG, tested three more devices in the same population. It tested the Watch BP device, which is a modified sphygmomanometer, and compared it with a 12-lead ECG. The resulting sensitivity was 96.34% (95% C.I. 89.68–99.24%) and the specificity was 93.45% (95% C.I. 90.25–95.85%). The same study tested two more devices that can detect AF by using heart rate variability. The Polar H7 device had a sensitivity of 96.34% (95% C.I. 89.68–99.24%) and specificity of 98.21% (95% C.I. 96.17–99.34%), and the Bodyguard 2 had a sensitivity of 96.34% (95% C.I. 89.68–99.24%) and a specificity of 98.51% (95% C.I. 96.56–99.52%).

The study of Reverberi et al., 2019 [58] is a diagnostic case–control study, which tested a chest-strap heart rate monitor in combination with the mobile application, RITMIA™, for the diagnosis of atrial fibrillation. The resulted sensitivity was 97.0% (95% C.I. 91.4–99.4%) and the specificity was 95.2% (95% C.I. 89.1–98.8%).

Finally, the study of Chen et al., 2020 [40], which was described in both the PPG and single-lead ECG groups, also tested the combination of both technologies. Specifically, during this test, the PPG mode was on, and if AF was detected, then participants were notified to perform a single-lead ECG. If the single-lead ECG was also positive for AF, then the result was considered positive. Otherwise, the final result was considered negative. The sensitivity for this mode was 80% (95% C.I. 72.52–85.90) and the specificity was 96.81% (95% C.I. 93.58–98.51).

## 4. Discussion

In this metanalysis, the two main technologies used to automatically detect AF (PPG and single-lead ECG) demonstrated very high diagnostic accuracy. Although the PPG technology proved to be more sensitive than the single-lead ECG, their 95% confidence intervals were overlapping. On the other hand, the two technologies had equal specificity.

In the PPG group, we noticed that four studies [31,33,36,42] showed significantly lower specificity compared to the rest (Figure 3). A further review of the studies demonstrated that, in most cases, the duration of the index test was prolonged, which may increase the false positive results. On contrary, the prolonged period of the index test can decrease the unclassified/unreadable results, since most of the studies with 0% unclassified/unreadable results used the devices for a longer period of time, and specifically from 10 min [35] to 1 week [41]. In the subgroup analysis between inpatients and outpatients in the PPG group, we did not observe any differences in the SROC curve; however, the small number of studies did not allow us to proceed to a quantitative synthesis.

In the single-lead ECG group, the lower pooled sensitivity could be partially explained by the lower duration of the index test. In most of the studies, it was applied for 30 to 60 s, compared to the PPG which was applied for at least 1 min. In addition, operation of a single-lead ECG requires action by the individual, and therefore unsupervised recordings could result in more poor-quality tracings. In this group, we performed two subgroup analyses. In the inpatients versus outpatients subgroup, the 95% confidence intervals were overlapping, and in the duration of the index test analysis (30 s vs. 60 s), we did not observe any clusters in the SROC curve. In relation to the unclassified/unreadable results, we observed significant heterogeneity in this group as well. Similarly with the PPG group, we noticed that most of the single-lead ECG studies with 0% unclassified/unreadable results used the index test for a prolonged period of time and/or allowed multiple measurements.

In both of the above groups, risk of bias was high or unknown in the participants selection domain, mainly due to case–control design in combination with ambiguity of the selection process. The rest of the domains were deemed mostly low risk of bias, and the applicability of the diagnostic test was satisfactory.

Other technologies, such as the modified sphygmomanometer and the heart rate variability, demonstrated very high sensitivity and specificity in their respective studies; however, the data were not enough to conduct a metanalysis. The study of Chen et al., 2020 [40] is especially interesting, because it tested the combination of PPG and single-lead ECG. During this study, individuals were being tested by continuous PPG, and they were asked to perform a single-lead ECG only when the PPG outcome was “possible AF”. Only if the single-lead ECG confirmed the diagnosis, then the individual was notified that they may suffer from AF. This study showed very high specificity but not as high sensitivity (~80%). Since more and more devices offer the possibility of both PPG and single-lead ECG, its combination can be proved valuable. All the technologies resulted in unclassified/unreadable results, which demonstrated significant heterogeneity among the studies.

Our findings are comparable with previous similar metanalyses [5,6,7,59] and suggest that widely available AI-based devices can accurately detect AF and can be used as a screening tool. So far, screening for AF is a controversial area. ESC guidelines support screening in targeted populations [3]; however, the American guidelines advise that the evidence is limited [60]. Long-term continuous screening in high-risk populations proved effective in detection of AF in a randomized study [61]. Another randomized trial showed that screening for AF led to fewer events for the combined primary outcome which included stroke, systemic embolism, bleeding leading to hospitalization and all-cause death [62]. Simulation studies using contemporary screening methods in elderly populations showed that screening is cost-effective, reduces stroke episodes, but increases bleeding risk and events [63,64].

In this context, our findings suggest that easily accessible AI-based devices can be convenient and non-invasive tools for AF screening. Compared to the traditional methods, these devices allow long-term passive monitoring, which is a paramount advantage given the paroxysmal and often asymptomatic nature of AF. Also, it provides individuals with the opportunity to record a trace at any time which can be useful when, for example, they develop symptoms. Most importantly, the AI-based devices do not require a health care professional at the stage of rhythm diagnosis; therefore, the devices allow more time for physicians to focus on the rest of the management.

## 5. Strengths and Limitations

Our study was designed and conducted according to the PRISMA guidelines. The review was very extended, since it identified almost 15,000 studies, and more than 1000 studies were reviewed based on their full text. The screening was conducted by two blinded and independent investigators, and the statistical analysis was performed for the two main technologies separately. Furthermore, we proceeded to subgroup analysis and described technologies other than the main two. We also calculated the false results for different prevalence values, which eventually is directly applicable to daily clinical practice.

On the other hand, our study demonstrates certain weaknesses. First of all, part of our study’s drawbacks arises from the limitations of the included studies. To start with the unclassified/unreadable results, there was significant heterogeneity among the studies. Many authors excluded them completely, some included them as false, and others included them as true or false depending on the reference standard. In our study, these results were excluded from the calculation of sensitivity and specificity and were described separately. Also, in many studies, atrial flutter and fibrillation were considered as the same disease, with the argument that their complications and treatment are very similar. However, others either excluded patients with atrial flutter completely or included them in the control group. Lastly, there was heterogeneity in the control groups, since some studies used only patients in sinus rhythm as control, and others used patients with any rhythm other than AF. Another issue was the use of multiple different devices and AI algorithms. On several occasions, the name or the version of the device and/or the AI algorithm were not even reported. Many authors tested the same devices with different algorithms, or they tested an amended version of the commercial algorithm. This heterogeneity constitutes a burden in the validation of devices and algorithms since it is difficult to appreciate the impact of their variability.

In addition, the executive part of our study appears to have certain limitations. First of all, the data extraction was performed mainly by one researcher, due to limited time and resources. Also, we had to amend our protocol, especially regarding the choice of reference standard test. Apart from the 12-lead ECG, we included other reference standard tests, since more tests are now accepted as gold standards for the diagnosis of atrial fibrillation. Furthermore, due to the complexity of diagnostic metanalysis, we could not proceed to more advanced statistical analyses, such as further subgroup and network metanalysis. Similarly, we did not calculate the reporting bias due to the complexity of metanalysis of diagnostic accuracy studies.

## 6. Conclusions

In summary, our findings support that both PPG and single-lead ECG devices have excellent sensitivity and specificity for the automated diagnosis of atrial fibrillation and can be used as screening tools. A prolonged period of monitoring may result in more false positive results, but less unclassified/unreadable outcomes. Further validation studies need to be conducted for alternative technologies, such as modified sphygmomanometry and combination of PPG and single-lead ECG. Further clinical trials are necessary to evaluate the cost-effectiveness, and risks and benefits, especially in younger populations where AI-based devices are widely available. 

## Figures and Tables

**Table 1:** Study characteristics (PPG, single-lead ECG and miscellaneous).

Author, Year	Setting	Study Design	Device	Algorithm	Time Used	Gold Standard	Age (Mean ± SD)	Sex (Females %)	N	N in AF Group	N in Control Group (Type of Control Group)	Group of AFL	TP	TN	FP	FN	Unclassified/ Uninterpretable
**Bacevicius et al., 2022 [39]**	Inpatients and outpatients	Case–control selected cross-sectional study	Prototype of the wearable device	Automatic PPG-based algorithm (PPG)	2 min.	3-lead Holter	AF: 65.6 ± 11.2, SR: 67.3 ± 14.2	AF: 47.1% SR: 46.1%	344	121	223 (SR)	Excluded from study	114	216	7	7	Excluded
**Chang et al., 2022 [33]**	Outpatients	Cohort selected cross-sectional study	Garmin Forerunner 945 smartwatch	Garmin Forerunner 945 smartwatch algorithm (PPG)	24 h	3-lead Holter	All participants: 66.1 ± 12.6 AF: 69.3 ± 11.5 Non-AF: 62.0 ± 12.9	All participants: 36.5% AF: 30.4% Non-AF: 44.3%	200	112	88 (non-AF)	AF group	109	78	10	3	0%
**Chen et al., 2020 (A) [40]**	Inpatients and outpatients	Case–control selected cross-sectional study	Amazfit Health Band 1S	RealBeats Artificial Intelligence Biological Data Engine (Huami Technology) (PPG)	3 min.	12-lead ECG	AF: 70.4 ± 11.5 Non-AF: 59.3 ± 14.8	AF: females: 43.3% Non-AF: females: 52.6%	401	139	244 (non-AF)	Control group	132	242	2	7	4.5%
**Van Haelst et al., 2018 (A) [11]**	Outpatients	Case–control selected cross-sectional study	Fibricheck	Fibricheck algorithm (PPG)	3 min.	12-lead ECG	All participants: 77.3 ± 8.0 AF: 78.8 ± 8.0 No AF: 75.9 ± 7.9	All participants: 57.4% AF: 51.1% Non-AF: 63.3%	190	75	93 (non-AF)	AF group	73	83	10	2	11.6%
**Mol et al., 2020 [35]**	Inpatients	Case–control selected cross-sectional study	iPhone 8	Algorithm developed by Happitech (Amsterdam, The Netherlands) (PPG)	90 s	Continuous electrocardiography	All participants: 69 ± 9	All participants: 43%	257	149	108 (SR)	Excluded from study	139	101	2	3	4.7%
**Sun et al., 2022 [34]**	Outpatients	Case–control selected cross-sectional study	Industrial camera (FLIR BFLY-U3-03S2C-CS)	DCNN model (PPG)	10 min. max or as much as tolerated	12-lead ECG	All participants: 69.3 ± 13.0 AF: 74.3 ± 12.5 Non-AF: 67.8 ± 13.0	All participants: 46% AF: 51.4% Non-AF: 44.8%	453	105	348 (no-AF)	Control group	98	342	6	7	Excluded
**Tison et al., 2018 [30]**	Inpatients	Case–control selected cross-sectional study	Apple Watch	Optimized Cardiogram app (PPG)	20 min prior- and 20 min post-cardioversion	12-lead ECG	All participants: 66.1 ± 10.7	All participants: 16%	51	51	51 (SR)	Excluded from study	50	46	5	1	0%
**Väliaho et al., 2019 (A) [36]**	Inpatients	Case–control selected cross-sectional study	Empatica E4 wrist band	MATLAB^®^ software version R2017b using AFEvidence or COSEn (PPG)	5 min	3-lead Holter	AF: 72.0 ± 14.3 years SR: 54.5 ± 18.6 years	AF: 42.5% SR: 44.9%	213	106	107 (SR)	Excluded from study	102	105	2	4	Excluded
**Väliaho et al., 2019 (B) [36]**	Inpatients	Case–control selected cross-sectional study	Empatica E4 wrist band	MATLAB^®^ software version R2017b using AFEvidence or COSEn (PPG)	5 min	3-lead Holter	AF: 72.0 ± 14.3 years SR: 54.5 ± 18.6 years	AF: 42.5% SR: 44.9%	213	106	107 (SR)	Excluded from study	101	105	2	5	Excluded
**Väliaho et al., 2021 (A) [41]**	Inpatients	Case–control selected cross-sectional study	Empatica E4 wrist band	MATLAB^®^ software (version R2017b) with a novel autocorrelation (AC) feature (PPG)	1 min every 10 min, 20 min, 30 min, 60 min	3-lead Holter	AF: 77.1 ± 9.7 SR: 67.3 ± 15.8	AF: 46.1% SR: 55.7%	173	76	97 (SR)	Unclear	75	79	18	1	0%
**Väliaho et al., 2021 (B) [41]**	Inpatients	Case–control selected cross-sectional study	Empatica E4 wrist band	MATLAB^®^ software (version R2017b) with a novel autocorrelation (AC) feature (PPG)	1 min every 10 min, 20 min, 30 min, 60 min	3-lead Holter	AF: 77.1 ± 9.7 SR: 67.3 ± 15.8	AF: 46.1% SR: 55.7%	173	76	97 (SR)	Unclear	75	87	10	1	0%
**Väliaho et al., 2021 (C) [41]**	Inpatients	Case–control selected cross-sectional study	Empatica E4 wrist band	MATLAB^®^ software (version R2017b) with a novel autocorrelation (AC) feature (PPG)	1 min every 10 min, 20 min, 30 min, 60 min	3-lead Holter	AF: 77.1 ± 9.7 SR: 67.3 ± 15.8	AF: 46.1% SR: 55.7%	173	76	97 (SR)	Unclear	72	94	3	4	0%
**Väliaho et al., 2021 (D) [41]**	Inpatients	Case–control selected cross-sectional study	Empatica E4 wrist band	MATLAB^®^ software (version R2017b) with a novel autocorrelation (AC) feature (PPG)	1 min every 10 min, 20 min, 30 min, 60 min	3-lead Holter	AF: 77.1 ± 9.7 SR: 67.3 ± 15.8	AF: 46.1% SR: 55.7%	173	76	97 (SR)	Unclear	70	96	1	6	0%
**Chan et al., 2016 (A) [32]**	Outpatients	Cohort selected cross-sectional study	iPhone 4S	Cardiio Rhythm smartphone application (Cardiio Inc.) (PPG)	51.3 s	single-lead ECG	All participants: 68.4 ± 12.2	All participants: 53.2%	1013	28	985 (non-AF)	Excluded from study	26	963	22	2	0%
**Dörr et al., 2019 (A) [37]**	Inpatients	Case–control selected cross-sectional study	Gear Fit 2, Samsung	Heartbeats application (Preventicus GmbH, Jena, Germany) (PPG)	1 min, 3 min and 5 min	single-lead ECG	All participants: 76.4 ± 9.5 AF: 77.4 ± 9.1 SR: 75.6 ± 9.8	All participants: 44.3% SR: 46.1% AF: 42.2%	650	237	271 (SR)	Excluded from study	222	266	5	15	21.8%
**Dörr et al., 2019 (B) [37]**	Inpatients	Case–control selected cross-sectional study	Gear Fit 2, Samsung	Heartbeats application (Preventicus GmbH, Jena, Germany) (PPG)	1 min, 3 min and 5 min	single-lead ECG	All participants: 76.4 ± 9.5 AF: 77.4 ± 9.1 SR: 75.6 ± 9.8	All participants: 44.3% SR: 46.1% AF: 42.2%	650	204	243 (SR)	Excluded from study	191	235	8	13	31.2%
**Dörr et al., 2019 (C) [37]**	Inpatients	Case–control selected cross-sectional study	Gear Fit 2, Samsung	Heartbeats application (Preventicus GmbH, Jena, Germany) (PPG)	1 min, 3 min and 5 min	single-lead ECG	All participants: 76.4 ± 9.5 AF: 77.4 ± 9.1 SR: 75.6 ± 9.8	All participants: 44.3% SR: 46.1% AF: 42.2%	650	167	202 (SR)	Excluded from study	156	198	4	11	43.2%
**Lubitz et al., 2022 [31]**	Outpatients	Cohort selected cross-sectional study	Fitbit device	Fitbit app (PPG)	1 week	single-lead ECG	All participants *: ≥75 years: 9.7% 65–74 years: 33.2% 55–64 years: 37.4% 40–54 years: 16.6% 22–39 years: 6.1%	All participants: 48.2%	1057	340	717 (non-AF)	AF group	230	706	11	110	0%
**Badertscher et al., 2022 [42]**	-	Cohort selected cross-sectional study	Withings Scanwatch	Withings Scanwatch detection algorithm (single-lead ECG)	30 s	12-lead ECG	All participants: 67 (54–76 years)	All participants: 48%	319	34	285 (SR)	No comment	19	247	3	6	13.8%
**Bumgarner MD et al., 2018 [43]**	Inpatients	Case–control selected cross-sectional study	Kardia Band	Kardia Band detection algorithm (single-lead ECG)	30 s	12-lead ECG	All participants: 68.2 ± 10.86	All participants: 17%	169	91	78 (SR)	In AF group	63	37	7	5	33.7%
**Campo et al., 2022 [44]**	Inpatients and outpatients	Case–control selected cross-sectional study	Withings Scanwatch	Withings Scanwatch detection algorithm (single-lead ECG)	30 s	12-lead ECG	All participants: 67.7 ± 14.8 AF: 74.3 ± 12.3 SR: 61.8 ± 14.3 Other arrhythmias: 66.9 ± 15.2 Unreadable ECGs: 78.8 ± 12.5	All participants: 61.1% AF: 42% SR: 34.5% Other arrhythmias: 40% Unreadable ECGs: 75%	258	87	155 (non-AF)	Control group	77	144	11	10	6.2%
**Chen et al., 2020 (B) [40]**	Inpatients and outpatients	Cohort selected cross-sectional study	Amazfit Health Band 1S	RealBeats Artificial Intelligence Biological Data Engine (Huami Technology) (single-lead ECG)	60 s	12-lead ECG	AF: 70.4 ± 11.5 Non-AF: 59.3 ± 14.8	AF: 43.3% Non-AF: 52.6%	401	150	25 (non-AF)	No comment	131	249	0	6	3.7%
**Cunha et al., 2020 [45]**	Inpatients	Cohort selected cross-sectional study	Kardia^®^ mobile	Kardia^®^ mobile algorithm (single-lead ECG)	30 s	12-lead ECG	-	-	129	22	78 (SR)	No comment	20	76	2	2	22.4%
**Desteghe et al., 2017 (A) [46]**	Inpatients	Cohort selected cross-sectional study	AliveCor	AliveCor algorithm (single-lead ECG)	30 s	12-lead ECG	All participants: 67.9 ± 14.6 AF: 73.1 ± 12.2 SR: 65.1 ± 15.0	All participants: 43.1% AF: 51.8% SR: 38.3%	265	22	243 (SR)	In AF group	12	237	6	10	0%
**Desteghe et al., 2017 (B) [46]**	Inpatients	Cohort selected cross-sectional study	MyDiagnostick	MyDiagnostick algorithm (single-lead ECG)	60 s	12-lead ECG	All participants: 67.9 ± 14.6 AF: 73.1 ± 12.2 SR: 65.1 ± 15.0	All participants: 43.1% AF: 51.8% SR: 38.3%	265	22	243 (SR)	In AF group	18	229	14	4	0%
**Desteghe et al., 2017 (C) [46]**	Inpatients	Cohort selected cross-sectional study	AliveCor	AliveCor algorithm (single-lead ECG)	30 s	6-lead ECG	-	-	113	19	94 (SR)	In AF group	15	92	2	4	0%
**Desteghe et al., 2017 (D) [46]**	Inpatients	Cohort selected cross-sectional study	MyDiagnostick	MyDiagnostick algorithm (single-lead ECG)	60 s	6-lead ECG	-	-	113	19	94 (SR)	In AF group	17	90	4	2	0%
**Ford et al., 2022 (A) [10]**	outpatients	Case–control selected cross-sectional study	Apple Watch 4	Apple Watch 4 algorithm (single-lead ECG)	30 s	12-lead ECG	All participants: 76 ± 7	All participants: 38%	125	31	94 (SR)	In AF group	6	76	0	6	29.6%
**Ford et al., 2022 (B) [10]**	outpatients	Case–control selected cross-sectional study	KardiaBand	KardiaBand algorithm (single-lead ECG)	30 s	12-lead ECG	All participants: 76 ± 7	All participants: 38%	125	31	94 (SR)	In AF group	26	68	5	1	20%
**Fu et al., 2021 (A) [47]**	-	Case–control selected cross-sectional study	Wearable Dynamic ECG Recorder	Amazfit CardiDoc application (single-lead ECG)	60 s	12-lead ECG	All participants: 59 ± 11.16 AF: 64.00 ± 9.38 SR:55.15 ± 11.01	All participants: 34% AF: 45.3% SR: 41%	114	53	61 (SR)	Excluded	47	61	0	4	1.8%
**Fu et al., 2021 (B) [47]**	-	Case–control selected cross-sectional study	Wearable Dynamic ECG Recorder	Amazfit CardiDoc application (single-lead ECG)	60 s	12-lead ECG	All participants: 59 ± 11.16 AF: 64.00 ± 9.38 SR:55.15 ± 11.01	All participants: 34% AF: 45.3% SR: 41%	114	53	61 (SR)	Excluded	50	61	0	2	0.9%
**Fu et al., 2021 (C) [47]**	-	Case–control selected cross-sectional study	Wearable Dynamic ECG Recorder	Amazfit CardiDoc application (single-lead ECG)	60 s	12-lead ECG	All participants: 59 ± 11.16 AF: 64.00 ± 9.38 SR:55.15 ± 11.01	All participants: 34% AF: 45.3% SR: 41%	114	53	61 (SR)	Excluded	50	61	0	2	0.9%
**Van Haelst et al., 2018 (B) [11]**	Outpatients	Cohort selected cross-sectional study	AliveCor	AliveCor algorithm (single-lead ECG)	30 s	12-lead ECG	All patients: 77.3 ± 8.0 AF: 78.8 ± 8.0 Non-AF: 75.9 ± 7.9	All participants: 57.4% AF: 51.1% No AF: 63.3%	190	75	93 (non-AF)	In AF group	73	83	10	2	19.3%
**Himmelreich JCL et al., 2019 [48]**	Outpatients	Cohort selected cross-sectional study	KardiaMobile	KardiaMobile (AliveCor, Inc.) algorithm (single-lead ECG)	30 s	12-lead ECG	All participants: 64.1 ± 14.7	All participants: 46.3%	214	23	191 (SR)	In AF group	20	187	4	0	1.4%
**Lown et al., 2018 (A) [49]**	Outpatients	Case–control selected cross-sectional study	AliveCor	AliveCor (Kardia version 4.7.0) algorithm (single-lead ECG)	30 s	12-lead ECG	All participants: 73.9 ± 6.1	-	418	82	336 (non-AF)	In AF group	72	332	2	2	2.4%
**Rajakariar et al., 2020 [50]**	Inpatients	Cohort selected cross-sectional study	KardiaBand	AliveCor Kardia application V.5.0.2 (AliveCor, Mountain View, CA, USA) (single-lead ECG)	30 s	12-lead ECG	All participants: 67 ± 16 AF: 76 ± 11 SR: 64 ± 17	All participants: 43.5% AF: 48% SR: 36%	200	38	162 (SR)	No comment	36	124	13	2	12.5%
**Santala et al., 2021 (1) [41]**	Inpatients	Case–control selected cross-sectional study	Suunto Movesense, Suunto, Vantaa, Finland (heart belt)	Awario, Heart2Save, Kuopio, Finland (single-lead ECG)	24 h	12-lead ECG	AF: 77 ± 10 SR: 68 ± 16	AF: 48% SR: 60%	159	73	86 (SR)	No comment	73	82	4	0	0%
**Santala et al., 2021 (2) (A) [51]**	Inpatients	Case–control selected cross-sectional study	single-lead Necklace-embedded ECG recorder (Including Movesense ECG-sensor, Suunto, Vantaa, Finland, Necklace-ECG)	Awario, Heart2Save, Kuopio, Finland (single-lead ECG)	30 s	3-lead Holter ECG	AF (years): 72.7 ± 14.1 SR (years): 61.5 ± 18.1	AF: 56.1% SR: 53.2%	145	66	79 (SR)	No comment	54	78	0	3	6.9%
**Santala et al., 2021 (2) (B) [51]**	Inpatients	Case–control selected cross-sectional study	single-lead Necklace-embedded ECG recorder (Including Movesense ECG-sensor, Suunto, Vantaa, Finland, Necklace-ECG)	Awario, Heart2Save, Kuopio, Finland (single-lead ECG)	30 s	3-lead Holter ECG	AF (years): 72.7 ± 14.1 SR (years): 61.5 ± 18.1	AF: 56.1% SR: 53.2%	145	66	79 (SR)	No comment	58	75	0	1	7.6%
**Santala et al., 2022 [52]**	Inpatients	Case–control selected cross-sectional study	Firstbeat Bodyguard 2, Firstbeat Technologies	Awario, Heart2Save (single-lead ECG)	24 h	3-lead Holter ECG	AF: 77 ± 10 SR: 68 ± 15	AF: 47% SR: 60%	178	79	99 (SR)	No comment	79	94	5	0	0%
**Abu-Alrub et al., 2022 (A) [53]**	-	Case–control selected cross-sectional study	Apple Watch Series 5^®^	Apple Watch Series 5^®^ (single-lead ECG)	30 s	12-lead ECG	All participants: 62 ± 7	All participants: 44%	200	100	100 (SR)	Excluded	87	86	1	7	9.5%
**Abu-Alrub et al., 2022 (B) [53]**	-	Case–control selected cross-sectional study	Samsung Galaxy Watch Active 3^®^	Samsung Galaxy Watch Active 3^®^ (single-lead ECG)	30 s	12-lead ECG	All participants: 62 ± 7	All participants: 44%	200	100	100 (SR)	Excluded	88	81	6	5	10%
**Abu-Alrub et al., 2022 (C) [53]**	-	Case–control selected cross-sectional study	Withings Move ECG^®^	Withings Move ECG^®^ algorithms (single-lead ECG)	30 s	12-lead ECG	All participants: 62 ± 7	All participants: 44%	200	100	100 (SR)	Excluded	78	80	3	2	18.5%
**Wegner et al., 2020 (A) [54]**	Inpatients	Cohort selected cross-sectional study	AliveCor Kardia ECG monitor	AliveCor Kardia ECG monitor algorithm (single-lead ECG)	30 s	12-lead ECG	All participants: 64 ± 15	All participants: 38.4%	92	27	65 (SR)	In AF group	19	45	8	0	21.7%
**Wegner et al., 2020 (B) [54]**	Inpatients	Cohort selected cross-sectional study	AliveCor Kardia ECG monitor	AliveCor Kardia ECG monitor algorithm (single-lead ECG)	30 s	12-lead ECG	All participants: 64 ± 15	All participants: 38.4%	92	27	65 (SR)	In AF group	15	39	3	0	38%
**William et al., 2018 [55]**	Inpatients	Case–control selected cross-sectional study	Kardia Mobile Cardiac Monitor	Kardia Mobile Cardiac Monitor (single-lead ECG)	30 s	12-lead ECG	All participants: 68.1 [42.6–85.6]	All participants: 32.7%	223	80	143 (SR)	In AF group	57	96	6	2	27.8%
**Chan et al., 2016 (B) [32]**	Outpatients	Cohort selected cross-sectional study	1st generation; AliveCor Inc.	AliveECG application (version 2.2.2) (single-lead ECG)	30 s	single-lead interpreted by cardiologists	All participants: 68.4 ± 12.2	All participants: 53.2%	1013	28 (AF)	985 (non-AF)	In control group	20	979	6	8	0%
**Dörr et al., 2019 (D) [37]**	Inpatients	Case–control selected cross-sectional study	AliveCor Kardia system	Heartbeats application (Preventicus GmbH, Jena, Germany) (single-lead ECG)	30 s	single-lead interpreted by cardiologists	All participants: 76.4 ± 9.5 AF: 77.4 ± 9.1 SR: 75.6 ± 9.8	All participants: 44.3% SR: 46.1% AF: 42.2%	650	319	331 (SR)	Excluded	279	262	7	1	15.5%
**Orchard et al., 2016 [56]**	Outpatients	Cohort selected cross-sectional study	AliveCor Heart Monitor	AliveCor Heart Monitor algorithm (single-lead ECG)	30 s	single-lead interpreted by cardiologists	-	-	972	38	934 (SR)	Excluded	36	844	8	2	8.4%
**Leńska-Mieciek et al., 2022 [57]**	Inpatients	Cohort selected cross-sectional study	Kardia Mobile portable device (AliveCor Inc., San Francisco, CA, USA)	AliveCor app. (single-lead ECG)	30 s	Single-lead ECG interpreted by cardiologist	All participants: 64.44 ± 10.52	All participants: 48%	50	1	49 (non-AF)	No comment	1	42	7	0	0%
**Lown et al., 2018 (B) [49]**	Outpatients	Case–control selected cross-sectional study	WatchBP	WatchBP algorithm (modified sphygmomanometer)	-	12-Lead ECG	All participants: 73.9 ± 6.1	-	418	82	336 (No AF)	In AF group	79	314	22	3	-
**Lown et al., 2018 (C) [49]**	Outpatients	Case–control selected cross-sectional study	Polar H7	PH7: (A Real-Time Atrial Fibrillation Detection Algorithm Based on the Instantaneous State of Heart Rate) (ECG data sensor)	-	12-Lead ECG	All participants: 73.9 ± 6.1	-	418	82	336 (No AF)	In AF group	79	330	6	3	-
**Lown et al., 2018 (D) [49]**	Outpatients	Case–control selected cross-sectional study	Bodyguard 2	BG2 (A Real-Time Atrial Fibrillation Detection Algorithm Based on the Instantaneous State of Heart Rate) (heart rate variability)	-	12-Lead ECG	All participants: 73.9 ± 6.1	-	418	82	336 (No AF)	In AF group	79	331	5	3	-
**Reverberi et al., 2019 [58]**	Inpatients	Case–control selected cross-sectional study	consumer-grade Bluetooth low-energy (BLE) HR monitor, of the chest-strap type	RITMIA™ (Heart Sentinel srl, Parma, Italy) (HR monitor)	-	12-lead ECG	All participants: 66.2 ± 10.7	All participants: 21.5%	182 **	99	83 (non-AF)	Excluded	96	79	4	3	-
**Chen et al., 2020 (C) [40]**	Inpatients and outpatients	Case–control selected cross-sectional study	Amazfit Health Band 1S (Huami Technology, Anhui, China)	RealBeats Artificial Intelligence Biological Data Engine (Huami Technology) (PPG combined with single-lead ECG)	-	12-lead ECG	AF: 70.4 ± 11.5 Non-AF: 59.3 ± 14.8	AF: 43.3% Non-AF: 52.6%	401	150	251 (non-AF)	Unclear	-	-	-	-	-

(N: number of participants; AF: Atrial Fibrillation; SR: Sinus Rhythm; AFL: Atrial Flutter; SD: standard deviation; * age distribution; ECG: electrocardiogram; DCNN: deep convolutional neural networks; rPPG: remote photoplethysmography; min: minutes; max: maximum; app: application; s: seconds; TP: true positive; TN: true negative; FP: false positive; FN: false negative; x (): median age (interquartile range); x []: average age [min. age–max. age]; Väliaho et al., 2019 (A): testing the AFEvidence algorithm; Chen et al., 2020 (A): testing PPG device; Chen et al., 2020 (B): testing single-lead ECG device; Chen et al. (C): testing combination of PPG and single-lead ECG; Väliaho et al., 2019 (B): testing the COSEn algorithm; Väliaho et al., 2021 (A): testing device performance when time interval between every measurement is 10 min; Väliaho et al., 2021 (B): testing device performance when time interval between every measurement is 20 min; Väliaho et al., 2021 (C): testing device performance when time interval between every measurement is 30 min; Väliaho et al., 2021 (D): testing device performance when time interval between every measurement is 60 min; Dörr et al., 2019 (A): testing performance of device when recording for 1 min; Dörr et al., 2019 (B): testing performance of device when recording for 3 min; Dörr et al., 2019 (C): testing performance of device when recording for 5 min; Dörr et al. (D): testing AliveCor; Desteghe et al., 2017 (A): testing the AliveCor in the cardiology ward population; Desteghe et al., 2017(B): testing the MyDiagnostick in the cardiology ward population; Desteghe et al., 2017 (C): testing the AliveCor in the geriatric ward population; Desteghe et al., 2017 (D): testing the MyDiagnostick in the geriatric ward population; Ford et al., 2022 (A): testing the Apple Watch 4; Ford et al., 2022 (B): testing the KardiaBand; Fu et al., 2021 (A): testing the device in supine position; Fu et al., 2021 (B): testing the device in upright position; Fu et al., 2021 (C): testing the device after individuals climbed to the 3rd floor; Van Haelst et al., 2018 (A): testing PPG device; Van Haelst et al., 2018 (B): testing single-lead ECG device; Santala et al., 2021 (1): published in October 2021; Santala et al., 2021 (2) (A): published in May 2021 and testing the device between the palms; Santala et al., 2021 (2) (B): published in May 2021 and testing the device in the chest; Abu-Alrub et al., 2022 (A): testing the Apple Watch 5; Abu-Alrub et al., 2022 (B): testing the Samsung Galaxy Watch Active 3; Abu-Alrub et al., 2022 (C): testing the Withings Move ECG; Wegner et al., 2020 (A): testing the lead I; Wegner et al., 2020 (B): testing the novel parasternal lead; Chan et al., 2016 (A): testing PPG device; Chan et al., 2016 (B): testing single-lead ECG device; Lown et al. (A): testing AliveCor; Lown et al., 2018 (B): testing the Watch BP; Lown et al., 2018 (C): testing the Polar H7 device; Lown et al., 2018 (D): testing the Bodyguard 2 device; ** N refers to ECGs before and after cardioversion.

## Data Availability

Data are contained within the article.

## References

[1] Freedman B., Potpara T.S., Lip G.Y.H. (2016). Stroke prevention in atrial fibrillation. Lancet.

[2] Mairesse G.H., Moran P., Van Gelder I.C., Elsner C., Rosenqvist M., Mant J., Banerjee A., Gorenek B., Brachmann J., Varma N. (2017). Screening for atrial fibrillation: A European Heart Rhythm Association (EHRA) consensus document endorsed by the Heart Rhythm Society (HRS), Asia Pacific Heart Rhythm Society (APHRS), and Sociedad Latinoamericana de Estimulación Cardíaca y Electrofisiología (SOLAECE). EP Eur..

[3] Hindricks G., Potpara T., Dagres N., Arbelo E., Bax J.J., Blomström-Lundqvist C., Boriani G., Castella M., Dan G.-A., Dilaveris P.E. (2021). 2020 ESC Guidelines for the diagnosis and management of atrial fibrillation developed in collaboration with the European Association for Cardio-Thoracic Surgery (EACTS): The Task Force for the diagnosis and management of atrial fibrillation of the European Society of Cardiology (ESC) Developed with the special contribution of the European Heart Rhythm Association (EHRA) of the ESC. Eur. Heart J..

[4] Li K.H.C., White F.A., Tipoe T., Liu T., Wong M.C., Jesuthasan A., Baranchuk A., Tse G., Yan B.P. (2019). The Current State of Mobile Phone Apps for Monitoring Heart Rate, Heart Rate Variability, and Atrial Fibrillation: Narrative Review. JMIR Mhealth Uhealth.

[5] Taggar J.S., Coleman T., Lewis S., Heneghan C., Jones M. (2015). Accuracy of methods for detecting an irregular pulse and suspected atrial fibrillation: A systematic review and meta-analysis. Eur. J. Prev. Cardiol..

[6] Yang T.Y., Huang L., Malwade S., Hsu C.-Y., Chen Y.C. (2021). Diagnostic Accuracy of Ambulatory Devices in Detecting Atrial Fibrillation: Systematic Review and Meta-analysis. JMIR Mhealth Uhealth.

[7] Nazarian S., Lam K., Darzi A., Ashrafian H. (2021). Diagnostic Accuracy of Smartwatches for the Detection of Cardiac Arrhythmia: Systematic Review and Meta-analysis. J. Med. Internet Res..

[8] Kavsaoğlu A.R., Polat K., Hariharan M. (2015). Non-invasive prediction of hemoglobin level using machine learning techniques with the PPG signal’s characteristics features. Appl. Soft Comput..

[9] Pereira T., Tran N., Gadhoumi K., Pelter M.M., Do D.H., Lee R.J., Colorado R., Meisel K., Hu X. (2020). Photoplethysmography based atrial fibrillation detection: A review. NPJ Digit. Med..

[10] Ford C., Xie C.X., Low A., Rajakariar K., Koshy A.N., Sajeev J.K., Roberts L., Pathik B., Teh A.W. (2022). Comparison of 2 Smart Watch Algorithms for Detection of Atrial Fibrillation and the Benefit of Clinician Interpretation. JACC Clin. Electrophysiol..

[11] Van Haelst R. (2016). The diagnostic accuracy of smartphone applications to detect atrial fibrillation: A head-to-head comparison between Fibricheck and AliveCor. Master Thesis.

[12] Benezet-Mazuecos J., García-Talavera C.S., Rubio J.M. (2018). Smart devices for a smart detection of atrial fibrillation. J. Thorac. Dis..

[13] Welcome to the Preferred Reporting Items for Systematic Reviews and Meta-Analyses (PRISMA) Website!. http://www.prisma-statement.org/.

[14] https://www.crd.york.ac.uk/prospero/display_record.php?RecordID=357232.

[15] https://www.cochrane.org/news/cochrane-recommends-covidence-new-reviews.

[16] PWhiting P.F., Rutjes A.W.S., Westwood M.E., Mallett S., Deeks J.J., Reitsma J.B., Leeflang M.M.G., Sterne J.A.C., Bossuyt P.M.M. (2011). QUADAS-2: A Revised Tool for the Quality Assessment of Diagnostic Accuracy Studies. Ann. Intern. Med..

[17] https://onlinelibrary.wiley.com/doi/full/10.1002/jrsm.1439.

[18] Zhou Y., Dendukuri N. (2014). Statistics for quantifying heterogeneity in univariate and bivariate meta-analyses of binary data: The case of meta-analyses of diagnostic accuracy. Stat. Med..

[19] Shen Q., Li J., Cui C., Wang X., Gao H., Liu C., Chen M. (2021). A wearable real-time telemonitoring electrocardiogram device compared with traditional Holter monitoring. J. Biomed. Res..

[20] Selder J.L., Breukel L., Blok S., van Rossum A.C., Tulevski I.I., Allaart C.P. (2019). A mobile one-lead ECG device incorporated in a symptom-driven remote arrhythmia monitoring program. The first 5982 Hartwacht ECGs. Neth. Heart J..

[21] Koh K.T., Law W.C., Zaw W.M., Foo D.H.P., Tan C.T., Steven A., Samuel D., Fam T.L., Chai C.H., Wong Z.S. (2021). Smartphone electrocardiogram for detecting atrial fibrillation after a cerebral ischaemic event: A multicentre randomized controlled trial. EP Eur..

[22] Hiraoka D., Inui T., Kawakami E., Oya M., Tsuji A., Honma K., Kawasaki Y., Ozawa Y., Shiko Y., Ueda H. (2022). Diagnosis of Atrial Fibrillation Using Machine Learning with Wearable Devices after Cardiac Surgery: Algorithm Development Study. JMIR Form. Res..

[23] Avram R., Ramsis M., Cristal A.D., Nathan V., Zhu L., Kim J., Kuang J., Gao A., Vittinghoff E., Rohdin-Bibby L. (2021). Validation of an algorithm for continuous monitoring of atrial fibrillation using a consumer smartwatch. Heart Rhythm..

[24] Scholten J., Jansen W.P., Horsthuis T., Mahes A.D., Winter M.M., Zwinderman A.H., Keijer J.T., Minneboo M., de Groot J.R., Bokma J.P. (2022). Six-lead device superior to single-lead smartwatch ECG in atrial fibrillation detection. Am. Heart J..

[25] Brasier N., Raichle C.J., Dörr M., Becke A., Nohturfft V., Weber S., Bulacher F., Salomon L., Noah T., Birkemeyer R. (2019). Detection of atrial fibrillation with a smartphone camera: First prospective, international, two-centre, clinical validation study (DETECT AF PRO). EP Eur..

[26] Palà E., Bustamante A., Clúa-Espuny J.L., Acosta J., González-Loyola F., Dos Santos S., Ribas-Segui D., Ballesta-Ors J., Penalba A., Giralt M. (2022). Blood-biomarkers and devices for atrial fibrillation screening: Lessons learned from the AFRICAT (Atrial Fibrillation Research In CATalonia) study. PLoS ONE.

[27] Rischard J., Waldmann V., Moulin T., Sharifzadehgan A., Lee R., Narayanan K., Garcia R., Marijon E. (2020). Assessment of Heart Rhythm Disorders Using the AliveCor Heart Monitor: Beyond the Detection of Atrial Fibrillation. JACC Clin. Electrophysiol..

[28] Mannhart D., Lischer M., Knecht S., Lavallaz J.d.F.d., Strebel I., Serban T., Vögeli D., Schaer B., Osswald S., Mueller C. (2023). Clinical Validation of 5 Direct-to-Consumer Wearable Smart Devices to Detect Atrial Fibrillation: BASEL Wearable Study. JACC Clin. Electrophysiol..

[29] Lau J.K., Lowres N., Neubeck L., Brieger D.B., Sy R.W., Galloway C.D., Albert D.E., Freedman S.B. (2013). iPhone ECG application for community screening to detect silent atrial fibrillation: A novel technology to prevent stroke. Int. J. Cardiol..

[30] Tison G.H., Sanchez J.M., Ballinger B., Singh A., Olgin J.E., Pletcher M.J., Vittinghoff E., Lee E.S., Fan S.M., Gladstone R.A. (2018). Passive Detection of Atrial Fibrillation Using a Commercially Available Smartwatch. JAMA Cardiol..

[31] Lubitz S.A., Faranesh A.Z., Selvaggi C., Atlas S.J., McManus D.D., Singer D.E., Pagoto S., McConnell M.V., Pantelopoulos A., Foulkes A.S. (2022). Detection of Atrial Fibrillation in a Large Population Using Wearable Devices: The Fitbit Heart Study. Circulation.

[32] Chan P., Wong C., Poh Y.C., Pun L., Leung W.W., Wong Y., Wong M.M., Poh M., Chu D.W., Siu C. (2016). Diagnostic Performance of a Smartphone-Based Photoplethysmographic Application for Atrial Fibrillation Screening in a Primary Care Setting. J. Am. Heart Assoc..

[33] Chang P.-C., Wen M.-S., Chou C.-C., Wang C.-C., Hung K.-C. (2022). Atrial fibrillation detection using ambulatory smartwatch photoplethysmography and validation with simultaneous holter recording. Am. Heart J..

[34] Sun Y., Yang Y.-Y., Wu B.-J., Huang P.-W., Cheng S.-E., Chen C.-C. (2022). Contactless facial video recording with deep learning models for the detection of atrial fibrillation. Sci. Rep..

[35] Mol D., Riezebos R.K., Marquering H.A., Werner M.E., Lobban T.C., de Jong J.S., de Groot J.R. (2020). Performance of an automated photoplethysmography-based artificial intelligence algorithm to detect atrial fibrillation. Cardiovasc. Digit. Health J..

[36] Väliaho E.-S., Kuoppa P., A Lipponen J., Martikainen T.J., Jäntti H., Rissanen T.T., Kolk I., Castrén M., Halonen J., Tarvainen M.P. (2019). Wrist band photoplethysmography in detection of individual pulses in atrial fibrillation and algorithm-based detection of atrial fibrillation. EP Eur..

[37] Dörr M., Nohturfft V., Brasier N., Bosshard E., Djurdjevic A., Gross S., Raichle C.J., Rhinisperger M., Stöckli R., Eckstein J. (2019). The WATCH AF Trial: SmartWATCHes for Detection of Atrial Fibrillation. JACC Clin. Electrophysiol..

[38] Väliaho E.-S., Lipponen J.A., Kuoppa P., Martikainen T.J., Jäntti H., Rissanen T.T., Castrén M., Halonen J., Tarvainen M.P., Laitinen T.M. (2022). Continuous 24-h Photoplethysmogram Monitoring Enables Detection of Atrial Fibrillation. Front. Physiol..

[39] Bacevicius J., Abramikas Z., Dvinelis E., Audzijoniene D., Petrylaite M., Marinskiene J., Staigyte J., Karuzas A., Juknevicius V., Jakaite R. (2022). High Specificity Wearable Device with Photoplethysmography and Six-Lead Electrocardiography for Atrial Fibrillation Detection Challenged by Frequent Premature Contractions: DoubleCheck-AF. Front. Cardiovasc. Med..

[40] Chen E., Jiang J., Su R., Gao M., Zhu S., Zhou J., Huo Y. (2020). A new smart wristband equipped with an artificial intelligence algorithm to detect atrial fibrillation. Heart Rhythm..

[41] Santala E.O., Halonen J., Martikainen S., Jäntti H., Rissanen T.T., Tarvainen M.P., Laitinen T.P., Laitinen T.M., Väliaho E.-S., Hartikainen J.E.K. (2021). Automatic Mobile Health Arrhythmia Monitoring for the Detection of Atrial Fibrillation: Prospective Feasibility, Accuracy, and User Experience Study. JMIR Mhealth Uhealth.

[42] Badertscher P., Lischer M., Mannhart D., Knecht S., Isenegger C., Lavallaz J.D.F.d., Schaer B., Osswald S., Kühne M., Sticherling C. (2022). Clinical validation of a novel smartwatch for automated detection of atrial fibrillation. Heart Rhythm. O2.

[43] Bumgarner J.M., Lambert C.T., Hussein A.A., Cantillon D.J., Baranowski B., Wolski K., Lindsay B.D., Wazni O.M., Tarakji K.G. (2018). Smartwatch Algorithm for Automated Detection of Atrial Fibrillation. J. Am. Coll. Cardiol..

[44] Campo D., Elie V., de Gallard T., Bartet P., Morichau-Beauchant T., Genain N., Fayol A., Fouassier D., Pasteur-Rousseau A., Puymirat E. (2022). Atrial Fibrillation Detection With an Analog Smartwatch: Prospective Clinical Study and Algorithm Validation. JMIR Form. Res..

[45] Cunha S., Antunes E., Antoniou S., Tiago S., Relvas R., Fernandez-Llimós F., da Costa F.A. (2020). Raising awareness and early detection of atrial fibrillation, an experience resorting to mobile technology centred on informed individuals. Res. Soc. Adm. Pharm..

[46] Desteghe L., Raymaekers Z., Lutin M., Vijgen J., Dilling-Boer D., Koopman P., Schurmans J., Vanduynhoven P., Dendale P., Heidbuchel H. (2017). Performance of handheld electrocardiogram devices to detect atrial fibrillation in a cardiology and geriatric ward setting. EP Eur..

[47] Fu W., Li R. (2021). Diagnostic performance of a wearing dynamic ECG recorder for atrial fibrillation screening: The HUAMI heart study. BMC Cardiovasc. Disord..

[48] Himmelreich J.C., Karregat E.P., Lucassen W.A., van Weert H.C., de Groot J.R., Handoko M.L., Nijveldt R., Harskamp R. (2019). Diagnostic Accuracy of a Smartphone-Operated, Single-Lead Electrocardiography Device for Detection of Rhythm and Conduction Abnormalities in Primary Care. Ann. Fam. Med..

[49] Lown M., Yue A.M., Shah B.N., Corbett S.J., Lewith G., Stuart B., Garrard J., Brown M., Little P., Moore M. (2018). Screening for Atrial Fibrillation Using Economical and Accurate Technology (From the SAFETY Study). Am. J. Cardiol..

[50] Rajakariar K., Koshy A.N., Sajeev J.K., Nair S., Roberts L., Teh A.W. (2020). Accuracy of a smartwatch based single-lead electrocardiogram device in detection of atrial fibrillation. Heart.

[51] Santala O.E., Lipponen J.A., Jäntti H., Rissanen T.T., Halonen J., Kolk I., Pohjantähti-Maaroos H., Tarvainen M.P., Väliaho E., Hartikainen J. (2021). Necklace-embedded electrocardiogram for the detection and diagnosis of atrial fibrillation. Clin. Cardiol..

[52] Santala O.E., A Lipponen J., Jäntti H., Rissanen T.T., Tarvainen M.P., Laitinen T.P., Laitinen T.M., Castrén M., Väliaho E.-S., A Rantula O. (2022). Continuous mHealth Patch Monitoring for the Algorithm-Based Detection of Atrial Fibrillation: Feasibility and Diagnostic Accuracy Study. JMIR Cardio..

[53] Abu-Alrub S., Strik M., Ramirez F.D., Moussaoui N., Racine H.P., Marchand H., Buliard S., Haïssaguerre M., Ploux S., Bordachar P. (2022). Smartwatch Electrocardiograms for Automated and Manual Diagnosis of Atrial Fibrillation: A Comparative Analysis of Three Models. Front. Cardiovasc. Med..

[54] Wegner F.K., Kochhäuser S., Ellermann C., Lange P.S., Frommeyer G., Leitz P., Eckardt L., Dechering D.G. (2020). Prospective blinded Evaluation of the smartphone-based AliveCor Kardia ECG monitor for Atrial Fibrillation detection: The PEAK-AF study. Eur. J. Intern. Med..

[55] William A.D., Kanbour M., Callahan T., Bhargava M., Varma N., Rickard J., Saliba W., Wolski K., Hussein A., Lindsay B.D. (2018). Assessing the accuracy of an automated atrial fibrillation detection algorithm using smartphone technology: The iREAD Study. Heart Rhythm..

[56] Orchard J., Lowres N., Ben Freedman S., Ladak L., Lee W., Zwar N., Peiris D., Kamaladasa Y., Li J., Neubeck L. (2016). Screening for atrial fibrillation during influenza vaccinations by primary care nurses using a smartphone electrocardiograph (iECG): A feasibility study. Eur. J. Prev. Cardiol..

[57] Leńska-Mieciek M., Kuls-Oszmaniec A., Dociak N., Kowalewski M., Sarwiński K., Osiecki A., Fiszer U. (2022). Mobile Single-Lead Electrocardiogram Technology for Atrial Fibrillation Detection in Acute Ischemic Stroke Patients. J. Clin. Med..

[58] Reverberi C., Rabia G., De Rosa F., Bosi D., Botti A., Benatti G. (2019). The RITMIA^TM^ Smartphone App for Automated Detection of Atrial Fibrillation: Accuracy in Consecutive Patients Undergoing Elective Electrical Cardioversion. Biomed. Res. Int..

[59] (2019). Lead-I ECG Devices for Detecting Symptomatic Atrial Fibrillation Using Single Time Point Testing in Primary Care. www.nice.org.uk/guidance/dg35.

[60] Davidson K.W., Barry M.J., Mangione C.M., Cabana M., Caughey A.B., Davis E.M., Donahue K.E., Doubeni C.A., Epling J.W., Us Preventive Services Task Force (2022). Screening for Atrial Fibrillation: US Preventive Services Task Force Recommendation Statement. JAMA.

[61] Sanna T., Diener H.-C., Passman R.S., Di Lazzaro V., Bernstein R.A., Morillo C.A., Rymer M.M., Thijs V., Rogers T., Beckers F. (2014). Cryptogenic Stroke and Underlying Atrial Fibrillation. N. Engl. J. Med..

[62] Svennberg E., Friberg L., Frykman V., Al-Khalili F., Engdahl J., Rosenqvist M. (2021). Clinical outcomes in systematic screening for atrial fibrillation (STROKESTOP): A multicentre, parallel group, unmasked, randomised controlled trial. Lancet.

[63] Chen W., Khurshid S., Singer D.E., Atlas S.J., Ashburner J.M., Ellinor P.T., McManus D.D., Lubitz S.A., Chhatwal J. (2022). Cost-effectiveness of Screening for Atrial Fibrillation Using Wearable Devices. JAMA Health Forum.

[64] Khurshid S., Chen W., Singer D.E., Atlas S.J., Ashburner J.M., Choi J.G., Hur C., Ellinor P.T., McManus D.D., Chhatwal J. (2021). Comparative clinical effectiveness of population-based atrial fibrillation screening using contemporary modalities: A decision-analytic model. J. Am. Heart Assoc..

